# Supplementary figures and images for: Dynamic decoding and dual synthetic data for automatic correction of grammar in low-resource scenario
Source: PeerJ Comput Sci. 2024 Jul 5;10:e2122. doi: 10.7717/peerj-cs.2122 (PMC11232608; doi:10.7717/peerj-cs.2122)

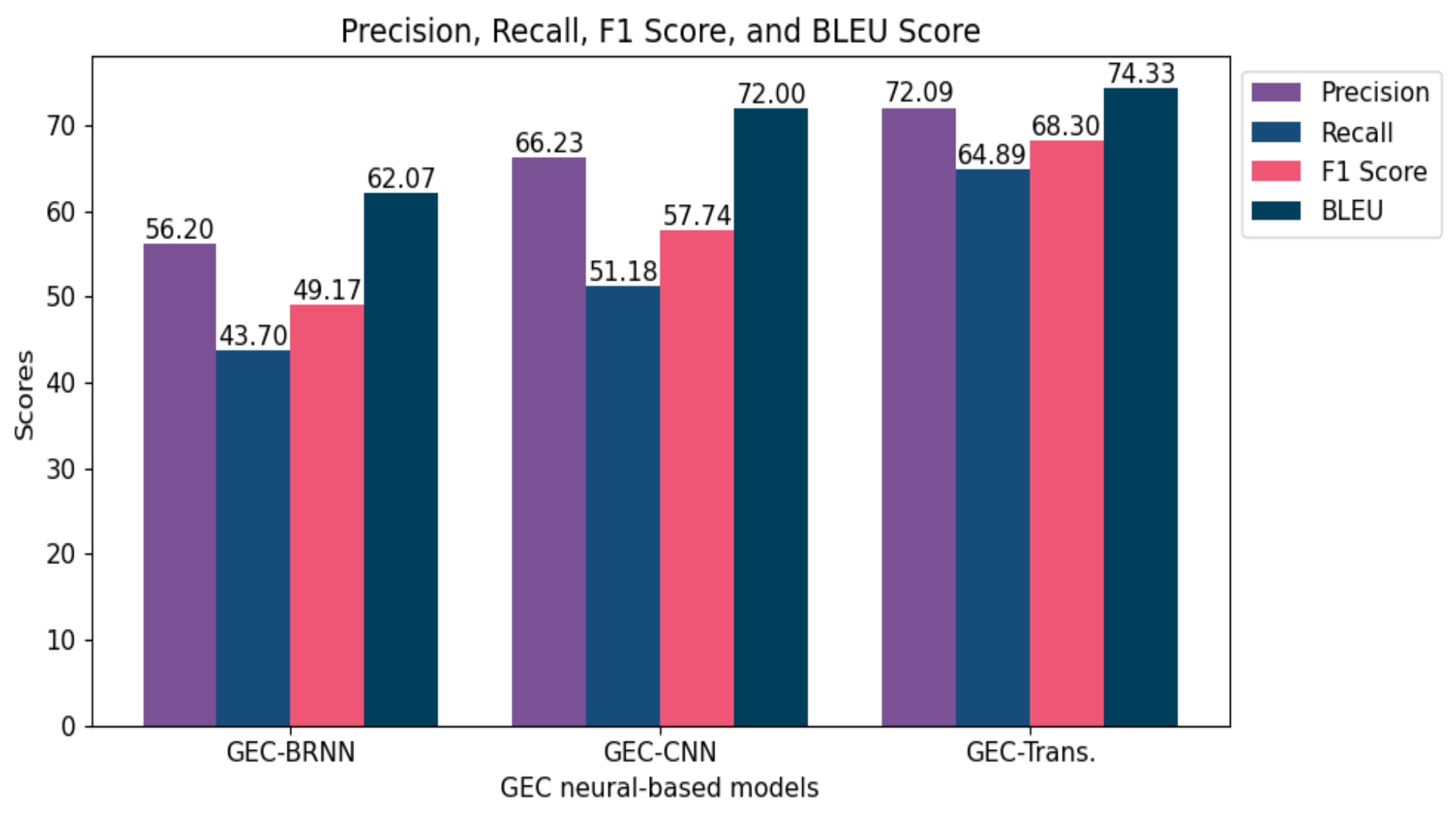

Supplement: Supplemental Information 1 [file peerj-cs-10-2122-s001.zip › SiaGEC-framework-SiaGEC/Figures and diagrams/cs-91671-R1.png]

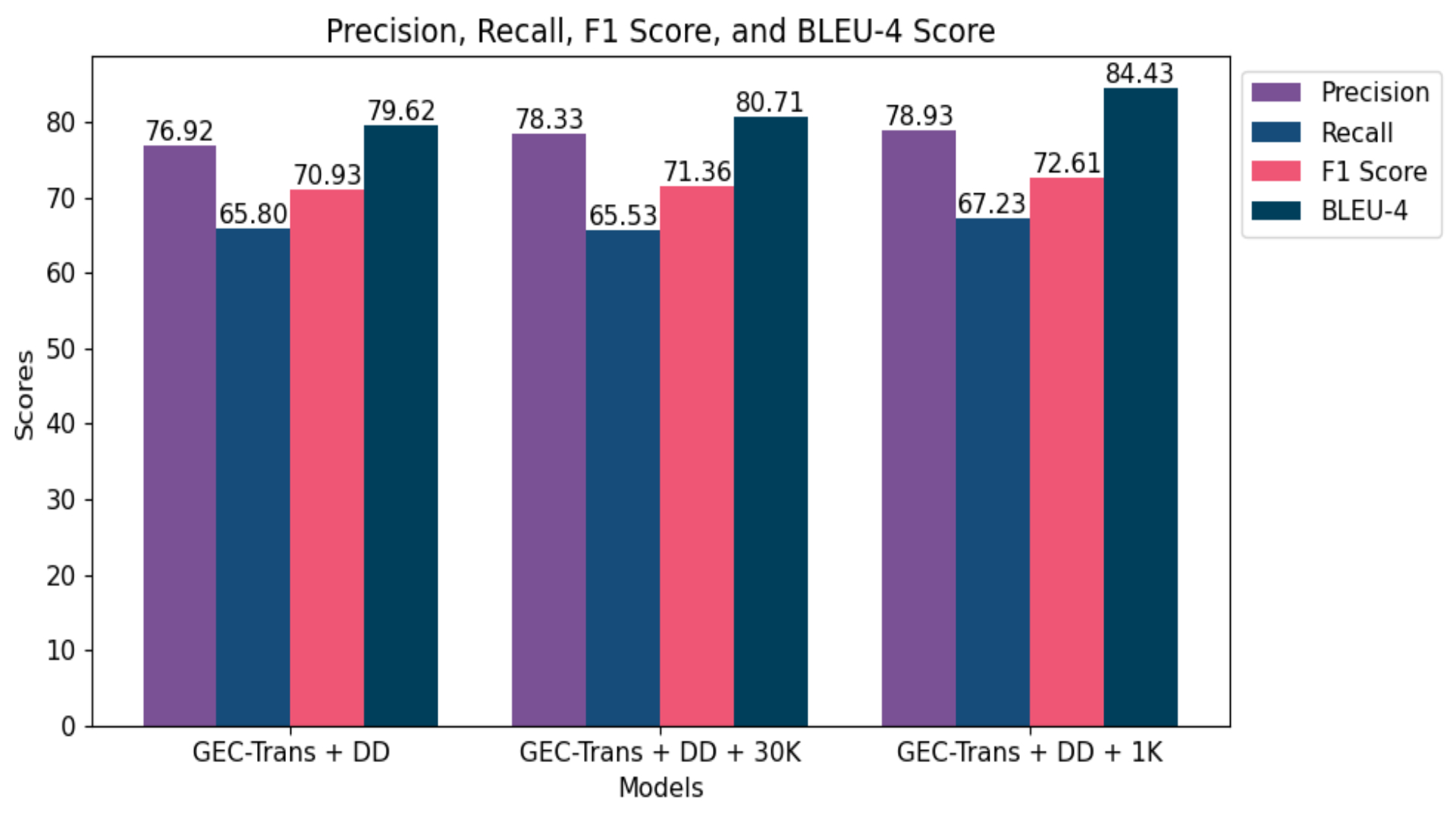

Supplement: Supplemental Information 1 [file peerj-cs-10-2122-s001.zip › SiaGEC-framework-SiaGEC/Figures and diagrams/cs-91671-R2.png]

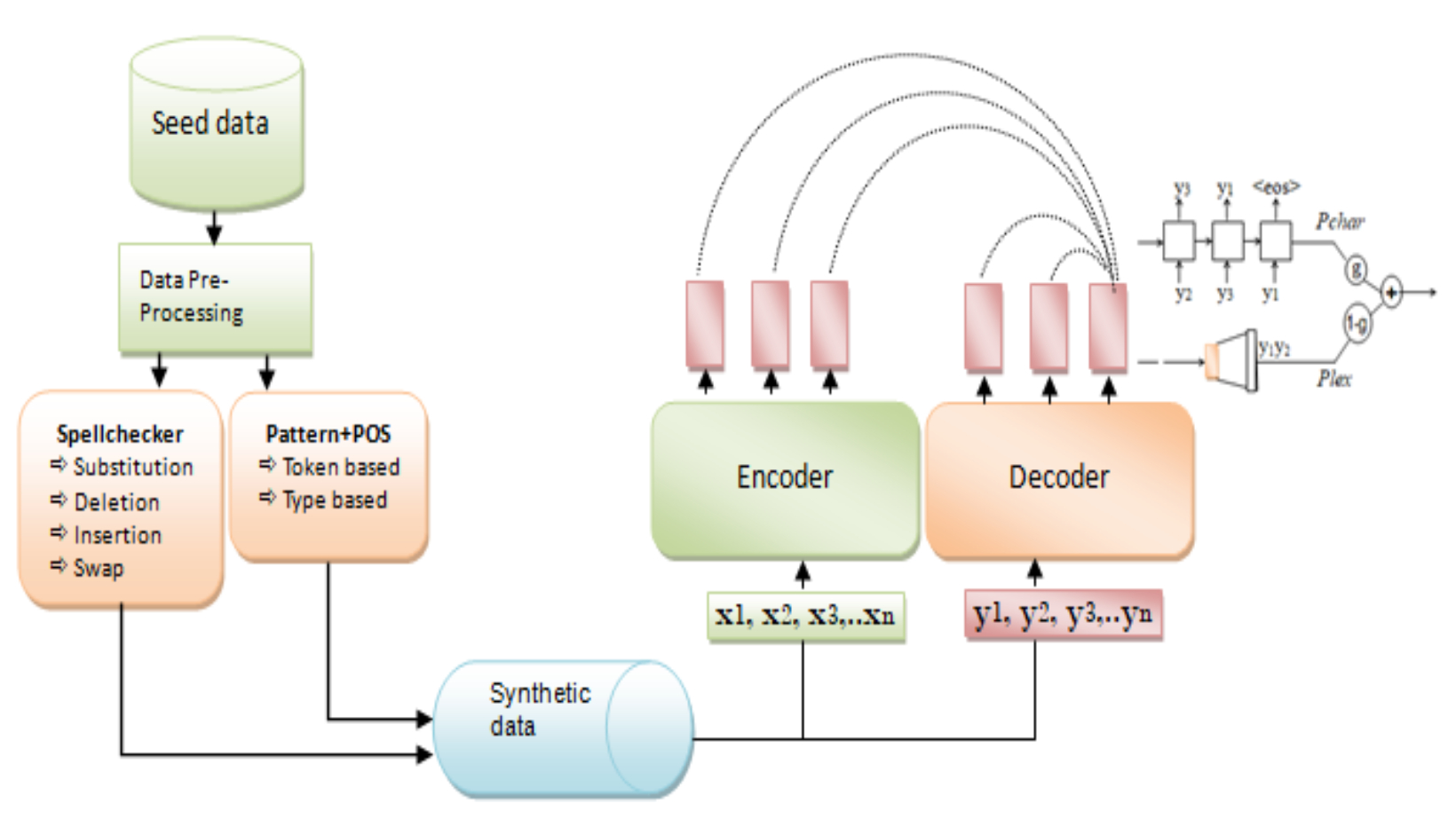

Supplement: Supplemental Information 1 [file peerj-cs-10-2122-s001.zip › SiaGEC-framework-SiaGEC/Figures and diagrams/cs-91671-figu1.png]

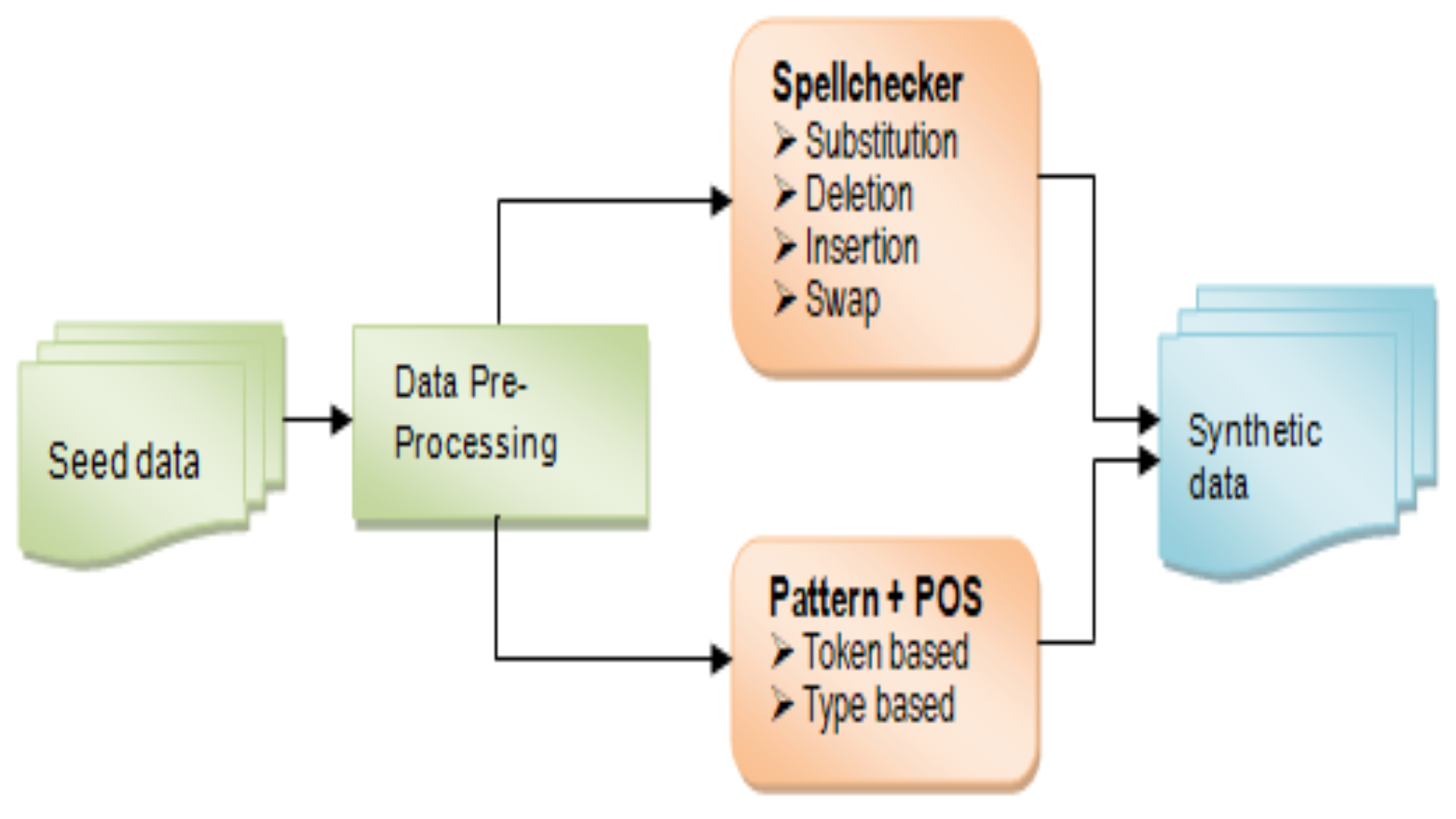

Supplement: Supplemental Information 1 [file peerj-cs-10-2122-s001.zip › SiaGEC-framework-SiaGEC/Figures and diagrams/cs-91671-figu2.png]

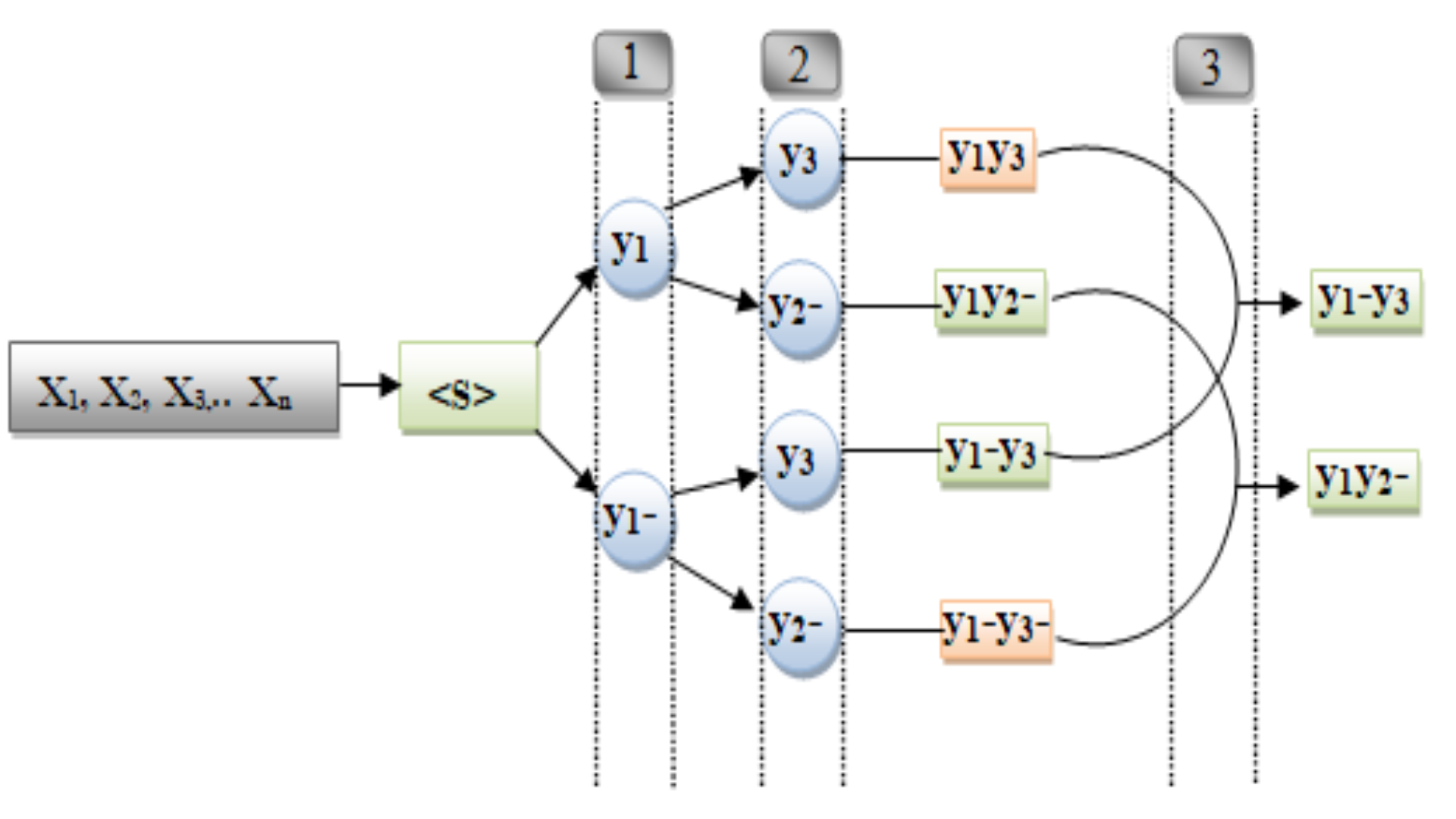

Supplement: Supplemental Information 1 [file peerj-cs-10-2122-s001.zip › SiaGEC-framework-SiaGEC/Figures and diagrams/cs-91671-figu3.png]
